# New Data on *Pterygodermatites* (*Pterygodermatites*) *plagiostoma* Wedl, 1861 (Nematoda, Rictulariidae) Parasite of the Algerian Hedgehog *Atelerix algirus* Linnaeus, 1758 (Eulipotyphla: Erinaceidae) from the Canary Islands †

**DOI:** 10.3390/ani12151991

**Published:** 2022-08-05

**Authors:** Jordi Miquel, Alexis Ribas, Román Pino-Vera, Elena Izquierdo-Rodríguez, Natalia Martín-Carrillo, Carlos Feliu, Pilar Foronda

**Affiliations:** 1 Secció de Parasitologia, Departament de Biologia, Sanitat i Medi Ambient, Facultat de Farmàcia i Ciències de l’Alimentació, Universitat de Barcelona, Avgda. Joan XXIII, sn, 08028 Barcelona, Spain; 2 Institut de Recerca de la Biodiversitat (IRBio), Universitat de Barcelona, Avgda. Diagonal, 645, 08028 Barcelona, Spain; 3 Departamento Obstetricia y Ginecología, Pediatría, Medicina Preventiva y Salud Pública, Toxicología, Medicina Legal y Forense y Parasitología, Facultad de Farmacia, Universidad de La Laguna, Avda. Astrofísico F. Sánchez, sn, 38203 La Laguna, Canary Islands, Spain; 4 Instituto Universitario de Enfermedades Tropicales y Salud Pública de Canarias, Universidad de La Laguna, Avda. Astrofísico F. Sánchez, sn, 38203 La Laguna, Canary Islands, Spain

**Keywords:** *Pterygodermatites* (*Pterygodermatites*) *plagiostoma*, Rictulariidae, *Atelerix algirus*, Erinaceidae, Canary Islands

## Abstract

**Simple Summary:**

A redescription of *Pterygodermatites* (*Pterygodermatites*) *plagiostoma* (Nematoda, Rictulariidae) is made by means of light and scanning electron microscopy, including the first data on male specimens. The morphologic study was based on specimens recovered from two Algerian hedgehogs from Tenerife and Gran Canaria islands (Canary Archipelago, Spain). The main differential characteristics of *P.* (*P.*) *plagiostoma* males are the number of cuticular projection pairs, the number of precloacal fans, and the size of spicules. The cloacal papillae are arranged according to the Ascaridida type, with two precloacal pairs, an unpaired precloacal papilla, one pair lateral to the cloaca, six postcloacal pairs, and a pair of phasmids. Females are mainly characterized and differentiated by the number of prevulvar pairs of cuticular projections, by the total number of cuticular projection pairs, by the level of differentiation from combs to spines and by the position of the vulva in relation to the esophagus–intestine junction. The comparison with species of the subgenus *P.* (*Pterygodermatites*) shows *P.* (*P.*) *plagiostoma* as a species clearly differentiated from the remaining species of this subgenus.

**Abstract:**

A redescription of the rictulariid nematode *Pterygodermatites* (*Pterygodermatites*) *plagiostoma* Wedl, 1861, is made by means of light and scanning electron microscopy, including the first data on male specimens. The morphologic study was based on specimens recovered from two Algerian hedgehogs (*Atelerix algirus*) from Tenerife and Gran Canaria islands (Canary Archipelago, Spain). The main characteristics of *P.* (*P.*) *plagiostoma* males are the presence of 49–53 pairs of cuticular projections, the presence of one or two midventral precloacal fans (generally one), and the size of two unequal spicules, measuring 98–123 µm (right spicule) and 185–236 µm (left spicule). The cloacal papillae are arranged according to the Ascaridida type. They include two precloacal pairs, an unpaired precloacal papilla, one pair lateral to the cloaca, six postcloacal pairs, and a pair of phasmids near the tail tip. Females are mainly characterized by the presence of 71–77 pairs of cuticular projections, with 43–46 pairs of prevulvar combs, by the differentiation from combs to spines at the level of or slightly posterior to the vulva and by the position of the vulva, located posteriorly to the esophagus–intestine junction. Clear differences were found between *P.* (*P.*) *plagiostoma* and related species of the subgenus *P.* (*Pterygodermatites*).

## 1. Introduction

*Pterygodermatites* (*Pterygodermatites*) *plagiostoma* is a rictulariid nematode frequently found parasitizing hedgehogs. It has been recorded in *Atelerix algirus*, *Erinaceus europaeus*, *Hemiechinus auritus*, and *Paraechinus aethiopicus* [1,2,3,4,5,6,7,8]. It has also been cited parasitizing the bat *Vespertilio mystacinus* (=*Myotis mystacinus*) (Vespertilionidae), the rodent *Sciurus melanogaster* (=*Callosciurus melanogaster*) (Sciuridae), and the carnivores *Vulpes vulpes niloticus* (Canidae) and *Paguma larvata* (Viverridae) [9,10,11,12]. However, the *P. plagiostoma* specimens of Parona [10] in *C. melanogaster* from Mentawei (Sumatra) were later studied by Jägerskiöld [3], who identified them as a new species *Rictularia fallax*, currently *P.* (*Mesopectines*) *fallax* after Quentin [13]. Moreover, the record of Sonsino [11] in the fox from Egypt was later considered *Rictularia affinis* (=*Pterygodermatites* (*Multipectines*) *affinis*) by Jägerskiöld [3]. The report of Willemoes-Suhm [12] in *M. mystacinus* from Germany, described as *Ophiostomum spinosum* and later identified as *Rictularia plagiostoma* [14,15], was finally considered as *P.* (*P.*) *spinosa* by Quentin [13]. Finally, the finding of Leiper [9] in the palm-civet *P. larvata* obtained from the London Zoo is probably a misidentification or an accidental parasitism [7] and, therefore, it must be considered with caution.

*Pterygodermatites* (*P.*) *plagiostoma* is the type species of both genus and nominal subgenus. The subgenus *P.* (*Pterygodermatites*) has only six species, namely *P.* (*P.*) *plagiostoma*, *P.* (*P.*) *aethechini*, *P.* (*P.*) *atlanticaensis*, *P.* (*P.*) *mexicana*, *P.* (*P.*) *shaldibini*, and *P.* (*P.*) *spinosa* [13,16,17,18]. In the original description of *P.* (*P.*) *plagiostoma*, Wedl [8] illustrates the caudal extremity of a male specimen showing two unequal spicules, but no other morphologic characteristic or measurements are presented. Despite the numerous posterior findings [1,2,3,4,5,6,7], to date, the detailed morphology of the male remains unknown [13,19].

The aim of the present study is to describe for the first time the male of *P.* (*P.*) *plagiostoma* and contribute with new data on females from a hedgehog *A. algirus* from the Canary Islands. In addition, the sequence of the mitochondrial cytochrome c oxidase subunit I gene (MT-CO1) of *P.* (*P.*) *plagiostoma* is provided.

## 2. Materials and Methods

### 2.1. Specimens

Two *Atelerix algirus* Linnaeus, 1758 (Eulipotyphla: Erinaceidae), were found dead on the road in El Rosario (Tenerife Island) on 3 June 2021 and in Jinámar (Gran Canaria Island) (Canary Archipelago, Spain) on 9 October 2019 and then they were scanned for intestinal helminths. A total of 149 rictulariid nematodes (38 males and 111 females) were found in the gastrointestinal tract of the two hedgehogs. They were identified as *P.* (*P.*) *plagiostoma* following the available literature [7,13].

### 2.2. Scanning Electron Microscopy Study

Six *P.* (*P.*) *plagiostoma* males and eight females were preserved for scanning electron microscopy (SEM) examination, fixed in 70% ethanol and posteriorly dehydrated in an ethanol series and critical point dried with carbon dioxide in an Emitech K850X (Quorum Technologies Ltd., Laughton, East Sussex, UK). Finally, specimens were mounted on stubs with conductive adhesive tape and colloidal silver, coated with carbon in an Emitech K950X (Quorum Technologies Ltd., Laughton, East Sussex, UK) evaporator, and examined using a Field Emission SEM JSM-7001F (Jeol) (Jeol Ltd., Tokyo, Japan) at 10 kV in the “Centres Científics i Tecnològics” of the University of Barcelona (CCiTUB).

### 2.3. Molecular Analyses

Genomic DNA samples were isolated from the mid-section fragment of *P.* (*P.*) *plagiostoma* following López et al. [20]. The DNA extraction procedure was checked using DeNovix DS-11+ Spectrophotometer (DeNovix Inc., Wilmington, DE, USA).

DNA amplification by PCR was conducted using the primer cocktail as described by Prosser et al. [21], for the barcode region of the mitochondrial cytochrome c oxidase subunit I gene (MT-CO1). The PCR amplification contained 1X Buffer (Bioline, London, UK), 0.2 mM of each dNTP (Bioline), 0.5 µL of each primer cocktail (10 µM of a three-forward-primers mix, and 10 µM of a three-reverse-primers mix), 1U of Taq DNA polymerase (Bioline), 1.5 mM MgCl_2_ (Bioline), and 20–30 ng of total genomic DNA in a total volume of 50 µL. Amplification was conducted with XP Cycler (Bioer Technology) using the following parameters: 94 °C for 1 min; five cycles at 94 °C for 40 s, 45 °C for 40 s, 72 °C for 1 min; followed by 35 cycles at 94 °C for 40 s, 51 °C for 40 s, 72 °C for 1 min; and a final extension at 72 °C for 5 min [21]. The resulting amplifications were visualized on 1.2% agarose gel at 100 V for 45 min.

The product of PCR was sequenced in Macrogen (Madrid, Spain) with primers NemF1_t1 and NemR1_t1 [21]. The analysis of the sequences was carried out with software MEGA X [22], using the multiple alignment program ClustalW included in MEGA X, and minor corrections were made by hand.

A phylogenetic analysis based on the MT-CO1 gene sequences of *P.* (*P.*) *plagiostoma* and other *Pterygodermatites* species available in GenBank was performed using Neighbor-Joining distance method (NJ) with the p-distance model [23] and Maximum-Likelihood (ML) method with Tamura–Nei model [24], both with at least 1000 bootstrap replications in MEGA X [22]. The sequence *Plectus aquatilis* KX017524 was used as the outgroup.

## 3. Results

### 3.1. Taxonomic Summary

Family Rictulariidae Hall, 1913.

Genus *Pterygodermatites* Wedl, 1861.

Subgenus *Pterygodermatites* (*Pterygodermatites*) Quentin, 1969.

**Figure 2:**
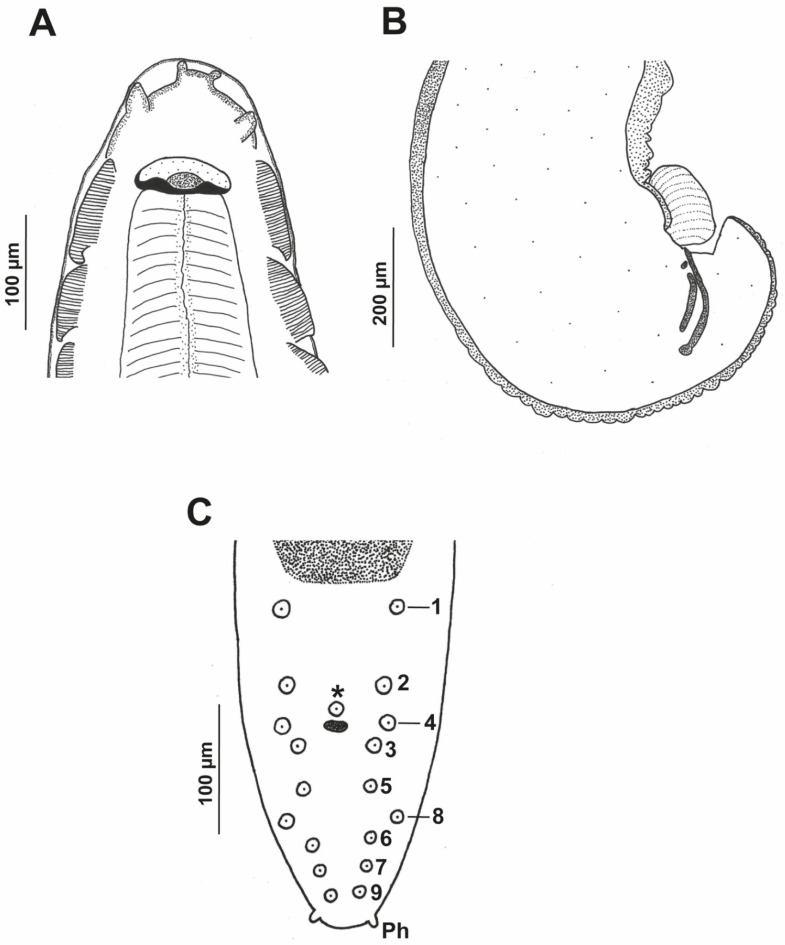
*Pterygodermatites* (*Pterygodermatites*) *plagiostoma* male. (**A**) Cephalic extremity, dorsal view. (**B**) Caudal extremity, lateral view. (**C**) Cloacal papillae. (*) Unpaired precloacal papilla; (Ph) phasmids.

**Figure 3:**
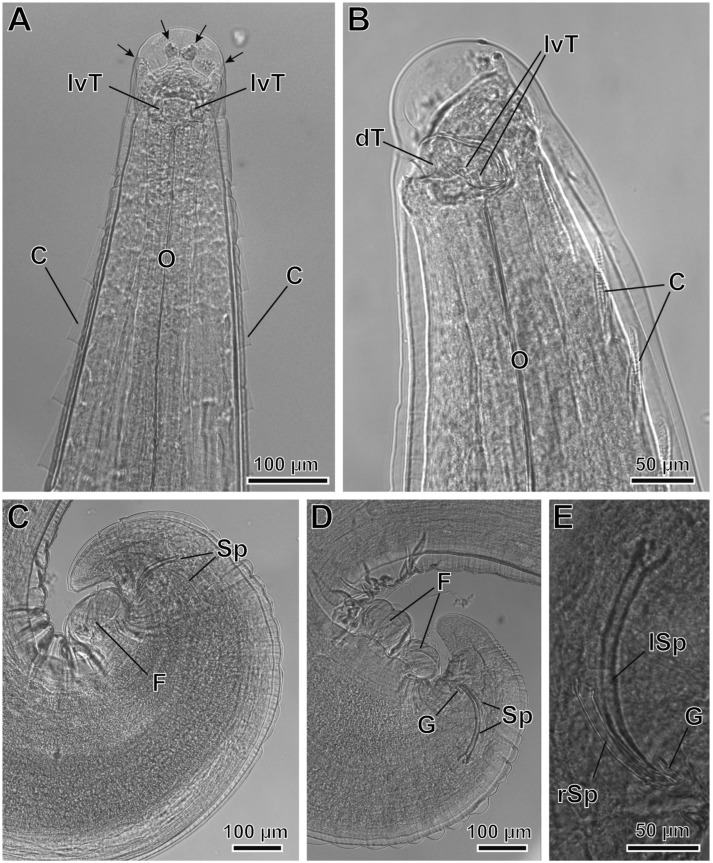
*Pterygodermatites* (*Pterygodermatites*) *plagiostoma* male, light microscopy. (**A**) Cephalic extremity, dorsal view showing the two lateroventral esophageal teeth (lvT) and four internal labial papillae (arrows). (**B**) Cephalic extremity, lateral view showing dorsal (dT) and lateroventral (lvT) esophageal teeth. (**C**) Caudal extremity of a male with one midventral fan (F). (**D**) Caudal extremity of a male with two midventral fans. (**E**) Detail of the right spicule (rSp), left spicule (lSp), and gubernaculum (G). (C) Cuticular combs; (O) esophagus; (Sp) spicules.

**Figure 4:**
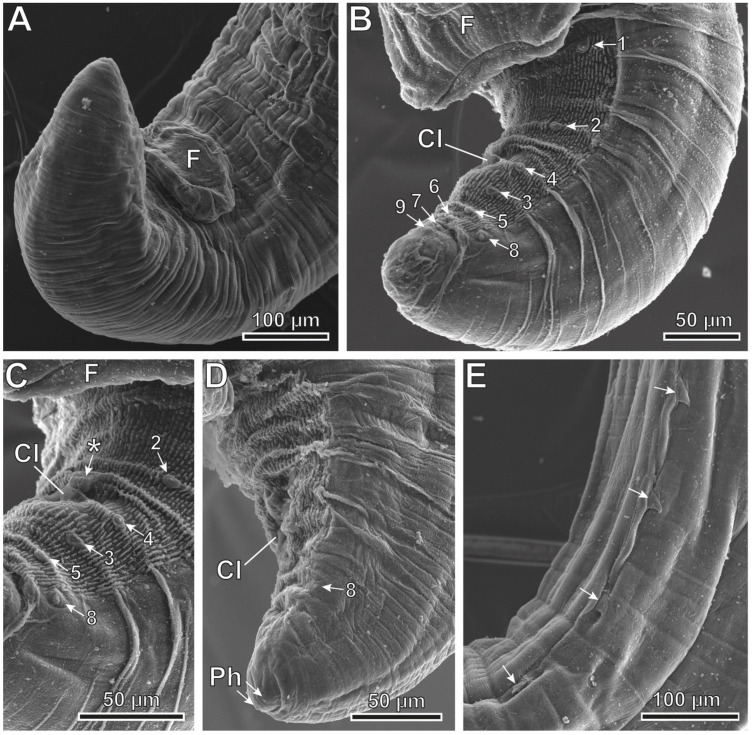
*Pterygodermatites* (*Pterygodermatites*) *plagiostoma* male’s caudal extremity, scanning electron microscopy. (**A**) Precloacal position of the midventral fan (F). (**B**) Lateral view showing the distribution cloacal papillae (pairs 1–9). (**C**) Cloacal region showing the unpaired precloacal papilla (*). (**D**) Position of phasmids (Ph) near the tail tip. (**E**) Lateral view illustrating the last cuticular spines (arrows). (Cl) Cloaca.

**Figure 5:**
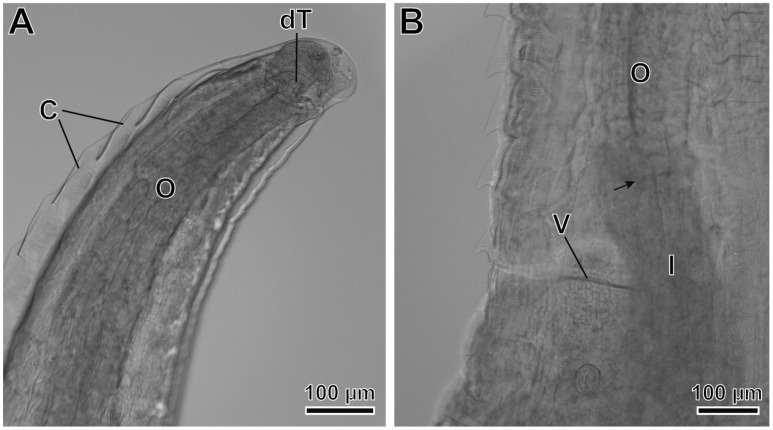
*Pterygodermatites* (*Pterygodermatites*) *plagiostoma* female, light microscopy. (**A**) Cephalic extremity, lateral view showing the dorsal esophageal tooth (dT). (**B**) Vulvar region. Note the posterior position of the vulva (V) in relation to the esophageal–intestinal junction (arrow). (C) Cuticular combs; (I) intestine; (O) esophagus.

**Figure 6:**
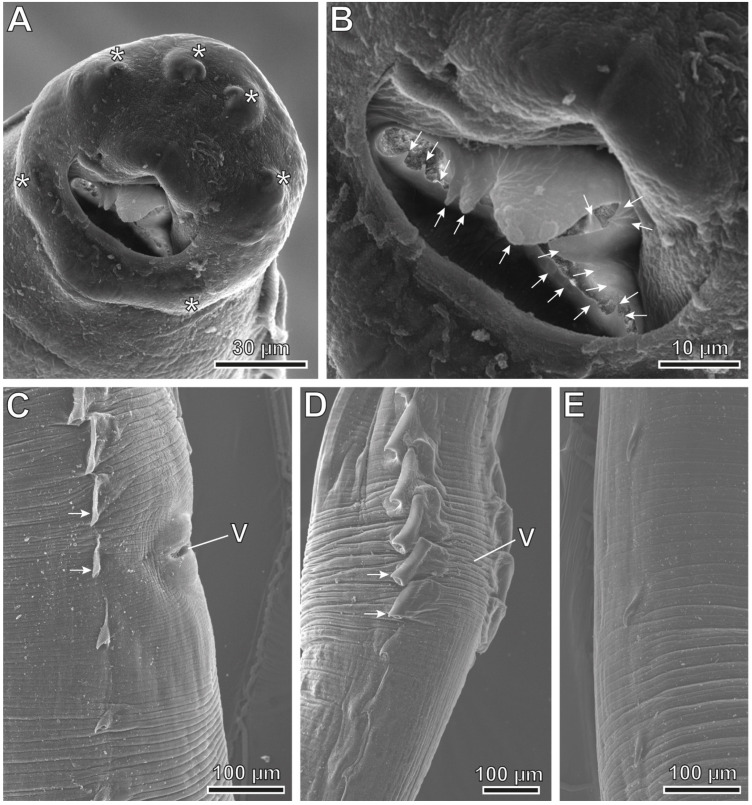
*Pterygodermatites* (*Pterygodermatites*) *plagiostoma* female, scanning electron microscopy. (**A**) Apical view showing the internal circle of labial papillae (*). (**B**) Detail of oral opening showing the irregular peribuccal denticles (arrows). (**C**,**D**) Vulvar region of two females showing the transition from combs to spines at a vulvar level or slightly posterior to the vulva (V), respectively. Note the presence of two reduced combs (arrows) before the appearance of spines. (**E**) Morphology of the most terminal spines.

*Pterygodermatites* (*Pterygodermatites*) *plagiostoma* Wedl, 1861 (Figures 2A–C, 3A–E, 4A–E, 5A,B and 6A–E).

Type host: *Atelerix algirus* Linnaeus, 1758 (Eulipotyphla: Erinaceidae).

Type locality: El Rosario (Tenerife Island, Canary Archipelago, Spain) (28°25′57.15″ N, 16°22′6.328″ W).

Other localities: Jinámar (Gran Canaria Island, Canary Archipelago, Spain) (28°1′58.457″ N, 15°25′8.994″ W).

Site of infection: small intestine.

Prevalence and intensity: two *A. algirus* studied with an intensity of 49 worms (15 males and 34 females) in the hedgehog from El Rosario and 100 worms (23 males and 77 females) in the hedgehog from Jinámar.

Type specimens: deposited in Muséum National d’Histoire Naturelle (Paris, France), under accession nos. MNHN HEL 1822 (8 males) and MNHN HEL 1823 (7 females).

Mitochondrial cytochrome c oxidase subunit I gene (MT-CO1) sequence: a fragment of 700 bp was obtained for the MT-CO1 of *P.* (*P.*) *plagiostoma* isolated from *A. algirus* in El Rosario, Tenerife. A 551 bp fragment was successfully sequenced and submitted to the GenBank database under the accession number ON502379.

### 3.2. Phylogenetic Tree

**Figure 1:**
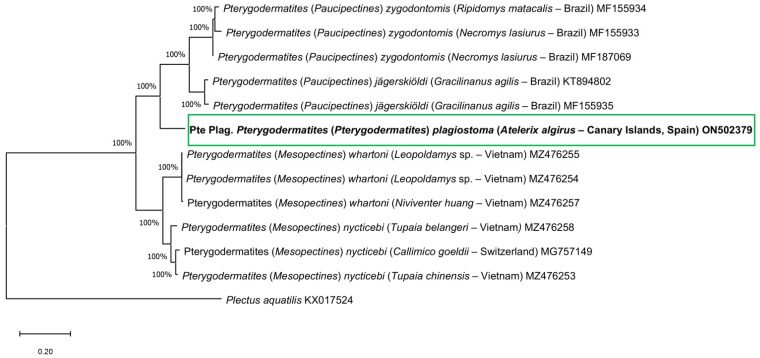
Phylogenetic analysis using the Maximum Likelihood method with p-distance and 1000 bootstrap replications based on the MT-CO1 gene sequences exploring the relationships among *Pterygodermatites* species including the nucleotide sequences obtained in this study (shown in bold). *Plectus aquatilis* was used as the outgroup.

The phylogenetic trees created using NJ and ML (Figure 1) methods based on the MT-CO1 gene showed similar results. *P.* (*P.*) *plagiostoma* is included in a clade together with *P.* (*Paucipectines*) *zygodontomis* and *P.* (*Pa.*) *jägerskiöldi* with a high bootstrap value (100%), and clearly separated from these two species. In the other clade *P.* (*Mesopectines*) *whartoni* and *P.* (*Me.*) *nycticebi* are included.

### 3.3. Description

General: medium-sized nematodes. Dorsal oral opening, surrounded by 6 internal labial papillae, 4 external pairs of cephalic papillae, and two lateral amphids (Figure 2A, Figure 3A and Figure 6A). Oral opening with irregularly distributed oral denticles of different sizes (Figure 4B); 3 internal esophageal teeth at the bottom of the buccal capsule, 1 dorsal and 2 lateroventral (Figure 2A, Figure 3A,B and Figure 5A). Well-developed buccal capsule. Two subventral rows of cuticular projections along the body, in the form of combs and spines both in males and females (Figure 3A,B, Figure 4E, Figure 5A and Figure 6C–E).

Male (17 specimens measured, mean in parentheses): Well-developed buccal capsule, oriented dorsally (Figure 2A and Figure 3B). Oral opening surrounded by irregular denticles. Presence of three esophageal teeth (Figure 2A and Figure 3A,B). Total pairs of combs 49–53 (50). Body length 5.36–7.67 mm (6.23 mm); width at the level of the esophagus basis 413–619 µm (498 µm). Esophagus length 2.09–2.87 mm (2.45 mm); width at base 111–180 µm (148 µm). Nerve ring located at 206–375 µm (269 µm) from the cephalic extremity; at the level of 3–4 pairs of cuticular combs. Deirids located at 516–795 µm (654 µm) from the cephalic extremity; at the level of 6–8 pairs of cuticular combs. Posterior end of body strongly curved ventrally (Figure 2B, Figure 3C,D and Figure 4A). Distance between the last cuticular spine and the tail tip 702–1060 µm (870 µm). The pericloacal surface is ornamented with cuticular markings (Figure 4B–D). Total of 19 caudal papillae which are sessile; 2 pairs of precloacal papillae (pairs 1 and 2), 1 unpaired precloacal papilla, 1 pair of papillae lateral to cloaca (pair 4), and 6 pairs of postcloacal papillae (pairs 3 and 5–9) (Figure 2C and Figure 4B,C). Pairs of papillae 1, 4, and 8 in a dorsolateral position, particularly the pair 8 that is located outside the ornamented area (Figure 2C and Figure 4B–D). A pair of phasmids near the tip of tail (Figure 2C and Figure 4D). One or two (generally one) precloacal midventral fans immediately before the first pair of precloacal papillae (Figure 2C, Figure 3C,D and Figure 4A,B). Spicules unequal in size; right spicule 98–123 µm (110 µm); left spicule 185–236 µm (217 µm) (Figure 2B and Figure 3C–E). Gubernaculum 28–39 µm (34 µm) (Figure 3E).

Female (17 gravid specimens measured, mean in parentheses): Well-developed buccal capsule, oriented dorsally (Figure 5A and Figure 6A). Oral opening surrounded by irregular denticles (Figure 6B). Presence of three esophageal teeth. Body length 12.07–17.54 mm (14.52 mm); width at the level of the vulva 423–712 µm (539 µm). Esophagus length 3.11–4.13 mm (3.71 mm); width at base 129–180 µm (154 µm). Nerve ring located at 285–463 µm (349 µm) from the cephalic extremity; at the level of 2–4 pairs of cuticular combs. Deirids located at 578–753 µm (644 µm) from the cephalic extremity; at the level of 6–9 pairs of cuticular combs. Total pairs of combs and spines 71–77 (74); prevulvar pairs of combs 43–46 (44); prevulvar combs in close contact to one another (Figure 5A,B and Figure 6C,D); at the level of vulva combs transform into two small pairs of combs and later into spines, and become more spaced from each other (Figure 5B and Figure 6C–E); postvulvar pairs of spines 28–32 (30); comb transformation to spines at the vulva or immediately posterior to the vulva (Figure 6C,D). Vulva located at 3.20–4.67 mm (3.99 mm) from the cephalic extremity; posterior to the esophagus–intestine junction (Figure 3B). Tail 177–350 µm (236 µm); with a terminal spine. Eggs oval, with a thick eggshell; embryonated 51.4–56.6 × 38.6–41.7 µm (55.6 × 42.1 µm); unembryonated 46.3–48.9 × 30.9–36.0 µm (47.5 × 33.1 µm).

## 4. Discussion

Within the subgenus *P.* (*Pterygodermatites*) there are only six described species (see Table 1). Considering male specimens, there is data for only three species, namely *P.* (*P.*) *aethechini*, *P.* (*P.*) *mexicana*, and *P.* (*P.*) *shaldybini* [13,16,17]. In the original description of *P.* (*P.*) *plagiostoma* [8], there was no additional data on males other than a caudal extremity showing the presence of two unequal spicules. In the present study, for the first time, we extensively describe the male of *P.* (*P.*) *plagiostoma*.

In the Rictulariidae, the number of cloacal papillae of males is constant (19 papillae arranged in nine pairs of papillae and an unpaired papilla [13]). Thus, there are two precloacal pairs, an unpaired precloacal papilla, either another precloacal pair or a pair located laterally to the cloaca and six postcloacal pairs. Additionally, near the tail tip there is a pair of phasmids. Quentin [13] analyzed the disposition of male cloacal papillae in numerous rictulariids and described three types of arrangement of papillae: the type Ascaridida presenting some pairs of papillae not aligned and slightly dorsolateral, the type Spirurida with all pairs of papillae aligned, and a third type with pedunculated and grouped papillae. In males of *P.* (*P.*) *plagiostoma*, the cloacal papillae arrangement corresponds to the type Ascaridida [13,27] having the pairs 1, 4, and 8 in a dorsolateral position, particularly the pair 8. According to Quentin [13], the type Ascaridida, which corresponds to the most primitive arrangement of cloacal papillae, is present in rictulariids included in both genera *Pterygodermatites* and *Rictularia*, e.g., *P.* (*Paucipectines*) *coloradensis*, *P.* (*Pa.*) *microti*, *P.* (*Pa.*) *ondatrae*, *P.* (*Pa.*) *zygodontomis*, *P.* (*Neopaucipectines*) *desportesi*, *R. citelli*, *R. dhanra*, *R. halli*, *R. lucifugus*, *R. macdonaldi*, and *R. proni*, among others. After the review of Quentin [13], the Ascaridida type of arrangement of cloacal papillae has been described in other species, particularly those belonging to the subgenus *P.* (*Paucipectines*), e.g., *P.* (*Pa.*) *andyraicola*, *P.* (*Pa.*) *argentinensis*, *P.* (*Pa.*) *baiomydis*, *P.* (*Pa.*) *chaetophracti*, and *P.* (*Pa.*) *hispanica* [28,29,30,31]. With respect to the subgenus *P.* (*Pterygodermatites*), Le Roux [17] reported only three precloacal and five postcloacal pairs of cloacal papillae in *P.* (*P.*) *aethechini*. However, the original description of Le Roux was illustrated with a lateral view of the male caudal extremity showing probably the unpaired precloacal papilla. In a posterior study concerning *P.* (*P.*) *mexicana*, Caspeta-Mandujano et al. [16] described in five sublateral pairs of cloacal papillae (pairs 1, 2, 4, 8, and 9) and four subventral pairs (pairs 3, 5, 6, and 7) but no unpaired precloacal papilla are mentioned.

Within the *P.* (*Pterygodermatites*) subgenus, males present three characters that are useful to discriminate between species: the number of cuticular projection pairs (combs and spines), the number of midventral fans, and the size of spicules [13,19,32]. Considering the species of the subgenus *P.* (*Pterygodermatites*) for which information on males is available, there are differences in the male of *P.* (*P.*) *plagiostoma* in the characters spicules size, number of fans, and number of cuticular projection pairs (see Table 1). In fact, in comparison to *P.* (*P.*) *aethechini* [17], the spicule sizes, particularly the right spicule is 98–123 µm in *P.* (*P.*) *plagiostoma* vs. 118–132 µm in *P.* (*P.*) *aethechini.* Additionally, in comparison to *P.* (*P.*) *mexicana* [16], *P.* (*P.*) *plagiostoma* presents a higher number of cuticular projection pairs (49–53 vs. 40 in *P.* (*P.*) *mexicana*), larger spicules (98–123 µm (right) and 185–236 µm (left) vs. 30–50 µm (right) and 83–111 µm (left) in *P.* (*P.*) *mexicana*) and lower number of midventral fans (1–2 vs. 3–4 in *P.* (*P.*) *mexicana*). Finally, in comparison to *P.* (*P.*) *shaldybini* [13], *P.* (*P.*) *plagiostoma* presents larger spicules (98–123 µm (right) and 185–236 µm (left in) vs. 70 µm (right) and 140 µm (left) in *P.* (*P.*) *shaldybini*).

Concerning females, the most discriminant characters between species are the prevulvar and total number of cuticular projection pairs, the body level where the transition from combs to spines occurs, and the position of the vulva in relation to the esophagus–intestine junction [13,19,32]. In fact, the differentiation of cuticular projections (from combs to spines) occurs at the level of the vulva in *P.* (*P.*) *plagiostoma* whereas it occurs at a posterior level in *P.* (*P.*) *aethechini* [17]. On the other hand, the vulva position is posterior to the esophagus–intestine junction in *P.* (*P.*) *plagiostoma* whereas it is anterior in *P.* (*P.*) *atlanticaensis* [18]. There is also a higher number of both prevulvar and total pairs of cuticular projections (combs and spines) (43–46/71–77) in *P.* (*P.*) *plagiostoma* in comparison to the number of these characters (40/66) in *P.* (*P.*) *mexicana* [16] although the number of cuticular projection pairs in *P.* (*P.*) *plagiostoma* (71–77) is surpassed by the 84 pairs described in *P.* (*P.*) *shaldybini* [13]. However, none of these discriminant characters of females differ between *P.* (*P.*) *plagiostoma* and *P.* (*P.*) *spinosa* [13].

*Pterygodermatites* (*P.*) *plagiostoma* is present in several hedgehog species (*A. algirus*, *E. europaeus*, *H. auritus*, and *P. aethiopicus*), and has a wide geographical distribution, including North Africa (Algeria, Tunisia and Egypt), Saudi Arabia, and Spain [1,2,3,4,5,6,7,8]. As for the remaining species of the subgenus, *P.* (*P.*) *atlanticaensis* and *P.* (*P.*) *mexicana* are parasites of bats in Brazil and Mexico [16,18], *P.* (*P.*) *spinosa* were detected in bats from Baviera (Germany) [12], *P.* (*P.*) *shaldybini* were found in bats and hedgehogs from Asia (Kazakstan, Turkmenistan, and Mongolia) [13,25,26], and *P.* (*P.*) *aethechini* were recorded in the hedgehog *Atelerix frontalis* from South Africa [16]. The present study enlarges the distribution of *P.* (*P.*) *plagiostoma* to the Tenerife and Gran Canaria islands (Canary Archipelago, Spain).

## 5. Conclusions

The present work on the rictulariid nematode *P.* (*P.*) *plagiostoma* contributes with the first morphoanatomical study of male specimens since its original description and provides further information on females. Moreover, the current finding in Tenerife and Gran Canaria islands (Canary Archipelago, Spain) enlarges the geographical distribution of this rictulariid.

The most useful characteristics to differentiate *P.* (*P.*) *plagiostoma* from the remaining species of the subgenus *P.* (*Pterygodermatites*) are the total number of pairs of cuticular projections, the size of spicules and the number of midventral fans for males, and the number of prevulvar and total cuticular projection pairs and the position of the vulva in relation to the esophagus–intestine junction for females. The parasitized hosts and the geographical distribution are also useful criteria. The arrangement of cloacal papillae in males of *P.* (*P.*) *plagiostoma* follows the type Ascaridida and this type seems to be a characteristic of the subgenera *P.* (*Pterygodermatites*), *P.* (*Paucipectines*), and *P.* (*Neopaucipectines*).

The analysis of available data on the species of *P.* (*Pterygodermatites*) emphasizes the need for more research, particularly in respect to male specimens, for which data are scarce or unknown for several species.

## Figures and Tables

**Table 1:** Main morphological characteristics, host group, and geographical distribution of the species of the subgenus *Pterygodermatites* (*Pterygodermatites*) Quentin, 1969.

Species	Males			Females			Host Group	Geographical Distribution	References
	CP	Spicule length right/left (in µm)	Fans	CP diff. *	VP #	Prevulvar CP/ Total CP			
*P.* (*P.*) *aethechini*	50	Unequal 118–132/190–225	1	Post	Post	42/75	Eulipotyphla	South Africa	[13,17]
*P.* (*P.*) *atlanticaensis*	–	–	–	Post	Ant	44–47/56–72	Chiroptera	Brazil	[18]
*P.* (*P.*) *mexicana*	40	Unequal 30–50/83–111	3–4	Post	Post	40/66	Chiroptera	Mexico	[16]
*P.* (*P.*) *plagiostoma*	49–53 ^1^	Unequal 98–123/185–236 ^1^	1–2 ^1^	Vulva-Post ^1^	Post ^1^	43–46/71–77 ^1^ 43–46/72–75 ^2^ 43–44/74–77 ^3^	Eulipotyphla Carnivora?	Saudi Arabia Mainland Spain Egypt Tunisia Algeria Eivissa Island (Spain) Canary Islands (Spain) London Zoo	[1] [2,7] [3,8,13] [4] [5] [6] [Present study] [9,13]
*P.* (*P.*) *shaldybini*	–	Unequal 70/140	–	–	–	42/84	Chiroptera Eulipotyphla	Kazakhstan Turkmenistan Mongolia	[13] [25] [26]
*P.* (*P.*) *spinosa*	–	–	–	–	–	43–44/77	Chiroptera	Germany	[12,13]

(*) Cuticular projections differentiation (combs to spines) in relation to the vulva; (#) position of vulva in relation to esophagus–intestine junction; (Ant) anterior; (CP) cuticular projections; (Post) posterior; (VP) position of the vulva; (–) unknown data; (?) doubtful data.^1^ Present study in *Atelerix algirus* from Tenerife and Gran Canaria islands (Canary Archipelago, Spain); ^2^ according to Miquel et al. [7] in *Erinaceus europaeus* from mainland Spain; ^3^ according to Jägerskiöld [3] in *Erinaceus auritus lybicus* (=*Hemiechinus auritus*) from Egypt.

## Data Availability

The type specimens are available upon request from MNHN Paris. Additional specimens are available upon request from the corresponding author.
